# Supplementary material for: The impact of a women-led community center on social cohesion in Tripoli, North Lebanon: A pilot randomized controlled trial
Source: PLoS One. 2026 Aug 5;21(8):e0336198. doi: 10.1371/journal.pone.0336198 (PMC13440862; doi:10.1371/journal.pone.0336198)
Supplement: Supplementary file 1 [file pone.0336198.s001.docx]

**S1 Appendix tables**

**Table A1: Correlates of attrition from the study (logistic regression results)** ^a, b^

| **Intervention group** | 1.1 |
| --- | --- |
|  | [0.43,2.78] |
| **Age** | 0.94* |
|  | [0.90,0.99] |
| **Did not previously know of center** | 0.45 |
|  | [0.15,1.32] |
| **Neighborhood (ref: Primarily Sunni)** |  |
| Primarily Alawite neighborhoods | 0.77 |
|  | [0.20,2.99] |
| Mixed neighborhoods | 0.65 |
|  | [0.21,1.95] |
| **Nationality (ref: Lebanese)** |  |
| Syrian | 0.87 |
|  | [0.30,2.53] |
| **Employment status (ref: employed)** |  |
| Unemployed | 4.57 |
|  | [0.54,38.55] |
| Not in Labor Force | 3.89 |
|  | [0.43,35.21] |
| **Not married** | 2.17 |
|  | [0.80,5.88] |
| Observations | 199 |

^a^ Exponentiated coefficients; 95% confidence intervals in brackets

^b^ * p<0.05, ** p<0.01, *** p<0.001

**Table A2: Balance between intervention and control groups at baseline (complete panel observations only)**

|  | **Control** | **Intervention** | **Total** | **p-value** *^a^* |
| --- | --- | --- | --- | --- |
|  | **N=85** | **N=92** | **N=177** |  |
| **Age (categorical)** |  |  |  | 0.22 |
| 18-34 | 26 (30.6%) | 30 (32.6%) | 56 (31.6%) |  |
| 35-64 | 59 (69.4%) | 59 (64.1%) | 118 (66.7%) |  |
| >=65 | 0 (0.0%) | 3 (3.3%) | 3 (1.7%) |  |
| **Age** | 40.1 (9.5) | 38.7 (11.3) | 39.4 (10.5) | 0.37 |
| **Nationality** |  |  |  | 0.69 |
| Lebanese | 53 (62.4%) | 60 (65.2%) | 113 (63.8%) |  |
| Syrian | 32 (37.6%) | 32 (34.8%) | 64 (36.2%) |  |
| **Neighborhood** |  |  |  | 0.12 |
| Primarily Sunni neighborhoods | 21 (25.0%) | 28 (30.8%) | 49 (28.0%) |  |
| Primarily Alawite neighborhoods | 17 (20.2%) | 27 (29.7%) | 44 (25.1%) |  |
| Mixed neighborhoods | 46 (54.8%) | 36 (39.6%) | 82 (46.9%) |  |
| **Marital status** |  |  |  | 0.95 |
| Married | 64 (76.2%) | 69 (75.8%) | 133 (76.0%) |  |
| Not married | 20 (23.8%) | 22 (24.2%) | 42 (24.0%) |  |
| **Employment status** |  |  |  | 0.21 |
| Employed | 16 (19.0%) | 13 (14.3%) | 29 (16.6%) |  |
| Unemployed | 35 (41.7%) | 50 (54.9%) | 85 (48.6%) |  |
| Not in Labor Force | 33 (39.3%) | 28 (30.8%) | 61 (34.9%) |  |
| **Heard of community center before** | |  |  | 0.33 |
| Yes | 39 (46.4%) | 49 (53.8%) | 88 (50.3%) |  |
| No | 45 (53.6%) | 42 (46.2%) | 87 (49.7%) |  |

*^a^ p-values are based on a Pearson’s chi-square test for categorical outcomes and a t-test for continuous outcomes.*

**Table A3: Correlates of never attending the intervention sessions (among women assigned to the intervention group, logistic regression results)** ^a, b^

| **Age** | 0.96 |
| --- | --- |
|  | [0.90,1.01] |
| **Did not previously know of center** | 0.55 |
|  | [0.15,2.00] |
| **Neighborhood (ref: Primarily Sunni)** |  |
| Primarily Alawite neighborhoods | 1.21 |
|  | [0.26,5.71] |
| Mixed neighborhoods | 1.12 |
|  | [0.31,4.10] |
| **Nationality (ref: Lebanese)** |  |
| Syrian | 1.04 |
|  | [0.30,3.53] |
| **Employment status (ref: employed)** |  |
| Unemployed | 3.47 |
|  | [0.62,19.50] |
| Not in Labor Force | 0.9 |
|  | [0.12,7.07] |
| **Not married** | 1.16 |
|  | [0.33,4.12] |
| Observations | 91 |

^a^ Exponentiated coefficients; 95% confidence intervals in brackets

^b^ * p<0.05, ** p<0.01, *** p<0.001

**Table A4: Sensitivity tests for the effect of the intervention on the social relationship scale (OLS regression results)** ^a, b^

|  | **(1)** | **(2)** | **(3)** |
| --- | --- | --- | --- |
|  | **Original coding (non-response coded to missing)** | **Non-response coded to lowest scale value** | **Non-response coded to median value** |
| Intervention | 0.60*** | 0.52*** | 0.51*** |
|  | [0.29,0.91] | [0.24,0.81] | [0.22,0.80] |
| Constant | -0.31** | -0.27* | -0.26* |
|  | [-0.54,-0.08] | [-0.47,-0.06] | [-0.47,-0.05] |
| Observations | 147 | 177 | 177 |

^a^ Exponentiated coefficients; 95% confidence intervals in brackets

^b^ * p<0.05, ** p<0.01, *** p<0.001

**Table A5: Local average treatment effect estimates for intervention effects (IV probit and IV regression results)** ^a, b^

|  | **(1)** | **(2)** | **(3)** | **(4)** | **(5)** | **(6)** | **(7)** | **(8)** | **(9)** | **(10)** | **(11)** |
| --- | --- | --- | --- | --- | --- | --- | --- | --- | --- | --- | --- |
|  | **Generalized trust (odds ratio)** | **Number of friends (cat)** | **No. friends different religion** | **No. friends different nationality** | **Social trust (3-item)** | **Social relationship scale** | **Civic engagement attitudes** | **Civic engagement behaviors** | **Civic engagement scale** | **GSE-3** | **WHO-5** |
|  |  |  |  |  |  |  |  |  |  |  |  |
| **Treated** | 1.46 | 0.17 | 0.11 | -0.04 | 0.19 | 1.03*** | 0.58* | 0.45^ | 0.58* | 0.15 | -3.31 |
|  | [0.71,3.00] | [-0.31,0.64] | [-0.40,0.62] | [-0.55,0.47] | [-0.19,0.58] | [0.50,1.57] | [0.09,1.08] | [-0.06,0.95] | [0.09,1.08] | [-0.31,0.60] | [-16.46,9.84] |
| Constant | 0.41*** | 2.64*** | 1.84*** | 1.83*** | 2.46*** | -0.52** | -0.28^ | -0.21 | -0.28^ | 3.49*** | 32.95*** |
|  | [0.37,0.45] | [2.36,2.91] | [1.55,2.13] | [1.54,2.12] | [2.23,2.70] | [-0.85,-0.19] | [-0.58,0.03] | [-0.50,0.07] | [-0.55,0.00] | [3.24,3.75] | [25.84,40.06] |
| **Obs.** | 177 | 176 | 168 | 165 | 168 | 147 | 177 | 177 | 177 | 177 | 177 |
